# The effect of metabolic syndrome on head and neck cancer incidence risk: a population-based prospective cohort study

**DOI:** 10.1186/s40170-021-00261-w

**Published:** 2021-06-03

**Authors:** Huaili Jiang, Lei Zhou, Qiangsheng He, Kanglun Jiang, Jinqiu Yuan, Xinsheng Huang

**Affiliations:** 1 Department of Otolaryngology, Zhongshan Hospital, Fudan University, Shanghai, China; 2 Cancer Center, Zhongshan Hospital, Fudan University, Shanghai, China; 3 Scientific Research Center, The Seventh Affiliated Hospital, Sun Yat-sen University, 628 Zhenyuan Road, Shenzhen, 518107 Guangdong China; 4 Center for Digestive Disease, The Seventh Affiliated Hospital, Sun Yat-sen University, Shenzhen, Guangdong China

**Keywords:** Metabolic syndrome, Metabolic syndrome components, Head and neck cancer, C-reactive protein

## Abstract

**Background:**

There are limited evidences clarifying the impact of metabolic syndrome (MS) and its components on head and neck cancer (HNC) incidence risk. We explored the correlation between MS, MS components, and the combined effects of MS and C-reactive protein (CRP) and HNC risk.

**Methods:**

This is a prospective analysis of 474,929 participants from the UK Biobank cohort. Cox proportional hazard regression was utilized to assess the hazard ratio (HR) and 95% confidence interval (CI) and to explore the non-linear correlation between an individual MS component and HNC risk.

**Results:**

Individuals with MS (HR, 1.05; 95%CI, 0.90–1.22) had no higher HNC risk than those without MS. More MS components showed no higher HNC risk. Nevertheless, hyperglycemia (HR, 1.22; 95%CI, 1.02–1.45) was independently correlated with elevated HNC risk. In a non-linear manner, waist circumference and high-density lipoprotein cholesterol (HDL-C) showed a U-shaped association with HNC risk. Further, piecewise linear model analysis indicated that higher male waist circumference, female waist circumference (≥93.16 cm), blood glucose (≥4.70 mmol/L) and male HDL-C (≥1.26mmo/L), and lower male HDL-C (<1.26mmo/L) were correlated with higher HNC risk. Increased CRP (≥1.00mg/dL) elevated HNC risk and individuals with MS and CRP≥1.00mg/dL had the highest HNC risk (HR, 1.29; 95%CI, 1.05–1.58). But no joint effect between MS and CRP was detected (p-interaction=0.501).

**Conclusions:**

MS are not correlated with elevated HNC risk. High waist circumference and blood glucose are independent risk factor of HNC incidence. Controlling HDL-C in an appropriate range can get the lowest risk of male HNC. No joint effect of MS and CRP exists in HNC tumorigenesis.

## Introduction

Head and neck cancer (HNC) constituted of 5% of all tumors [1]. Approximately 500,000 individuals are diagnosed each year, of which 350,000 cases die [2, 3]. Sixty percent of patients are already in advanced stage when being diagnosed [4, 5]. Although treatments including surgery, radiotherapy, and chemotherapy have been widely used, the 5-year survival rate of HNC is still only 50%, and the local recurrence rate is up to 50%, and the distant metastasis rate is 25% [6, 7]. Even if the treatment is successful, the patients may cause mental illness due to impaired vocalization, chewing, swallowing, respiratory function, and facial changes induced by surgery or radiotherapy. Studies have shown that among all HNC components, the suicide rate of patients with oral oropharyngeal cancer (53.1/100,000) and laryngeal cancer (46.8/100,000) second only to lung cancer (81.7/100,000) and stomach cancer (71.7/100,000), ranking third and fourth, respectively [8]. Therefore, early identification of risk factors is essential to reduce the morbidity and mortality of HNC.

Metabolic syndrome (MS) is a group of metabolic abnormalities, including hypertension, central obesity, elevated triglyceride, low high-density lipoprotein-cholesterol (HDL-C), and insulin resistance [9]. MS or its components are strongly correlated with cancer incidence risk and mortality. It has shown to increase the incidence of liver [10, 11], colorectal [12–14], pancreatic [15], endometrial [16], and breast cancer [17, 18]. MS component diabetes mellitus is also close associated to cancer risk [19], and abdominal obesity is notably correlated with higher risk and mortality of most common cancers [20]. The mechanism by which MS may influence cancer development is similar. The possible mechanisms of MS carcinogenesis are as follows: (1) hyperinsulinemia and insulin resistance, (2) chronic subclinical inflammation, (3) abnormal sex hormone metabolism, (4) injury induced by exposure of endocrine disruptors and air pollution, (5) chronic hyperglycemia, and (6) circadian rhythm disorder [21]. To our best knowledge, there is only one investigation showing inverse relations between MS and type 2 diabetes and HNC. However, to date, there have been no studies based on prospective analysis to explore the correlation between MS and HNC risk.

The UK Biobank recruited more than 500, 000 participants with an age from 37 to 73 years old recruited in UK from year 2006 to 2010. The UK Biobank documented beyond 2000 features, such as anthropometric measurements, sociodemographic assessments, clinical diagnosis, and self-reported behavioral outcomes, which provides us with a new chance to assess risk factors of cancer development in a large population-based samples. Based on the UK Biobank dataset, this study tried to clarify the major MS components connected to HNC, to explore possible non-linear correlations between its components and HNC, and to detect the mutual relations amongst MS, C-reactive protein (CRP), and HNC risk.

## Materials and methods

### Study design and participants

Our data application was approved by the UK Biobank on August 2019, and the application number was 51671. Detailed information on the research design and data collection methods of the UK Biobank cohort have been published [22]. We included all the UK Biobank participants who reported data on any measure of the MS components. The participants with any cancers being diagnosed before (*n* = 26868) were excluded (apart from non-melanoma skin cancer with a code of ICD-10 C44). For gestation will increase waist circumference and lead to potential metabolic changes, pregnant women (*n* = 149) were also excluded. The participants were followed until the date of HNC diagnosis or censoring. HNC was identified if participants were diagnosed as any of the following cancers: laryngeal cancer (ICD-10 C32), nasopharyngeal carcinoma (C11), tonsil cancer (C09), oropharyngeal cancer (C10), hypopharyngeal carcinoma (C12 and C13), nasal cavity and paranasal sinus cancer (C31), and oral cancer (C00-C06). Finally, 474,929 participants were involved in this study.

### Ethnic statement

The UK Biobank study was approved by the North West Multi-centre Research Ethics Committee, the England and Wales Patient Information Advisory Group, and the Scottish Community Health Index Advisory Group. All participants provided written informed consent before data collection.

### Data collection

Participants were invited to fill out a questionnaire during recruitment in their closest assessment center. Sociodemographic characteristics (i.e., age, gender, ethics, education, income levels), lifestyle information (i.e., physical activity, tobacco smoking, alcohol drinking), complications (i.e., diabetes mellitus, hypertension), and medicine intake information were collected. The International Physical Activity Questionnaire and the food frequency questionnaire were utilized to evaluate physical activity and diet intake respectively, which has been verified in a previous study [23]. Right arm diastolic and systolic blood pressure (DBP and SBP) was measured twice using an electronic sphygmomanometer and the average value was used. After normal exhalation, a Wessex non-stretchable spring tape measure was utilized to measure the waist circumference (cm) at the level of the umbilicus twice [24]. Plasma concentration of HDL-C, glucose, and triglycerides were tested utilizing a Beckman Coulter AU5800 analyzer. The baseline of CRP concentration was quantified utilizing the immuno-turbidimetric method.

### Outcome assessment

HNC cases were recognized by establishing links with the Health and Social Care Information Centre (located in England and Wales) and the Cancer and Death Registry of the National Health Service Central Register (located in Scotland). For more details about the linking process, please visit https://biobank.ndph.ox.ac.uk/showcase/refer.cgi?id=115558. The person-years were calculated recruitment date to any dates of death, the first HNC, or the end of follow-up (October 30, 2015).

### Definition for MS and MS components

We used the definition criteria of MS and its components developed by the American Heart Association/National Heart, Lung, and Blood Institute (AHA/NHLBI) [9]. Individuals with body mass index (BMI) greater than 30 kg/m^2^, or the waist circumference more than the values of cut points that are population- and country-specific definitions was diagnosed as central obesity [9]. Dyslipidemia for triglycerides (TG) was defined as plasma TG concentration ≥ 1.7 mmol/L (150 mg/dL) or currently on medications for hypertriglyceridemia [9]. HDL-C < 1.0 mmol/L (40 mg/dL) for males and < 1.30 mmol/L (50 mg/dL) for females or specific treatment for previously detected decreased HDL-C was defined as dyslipidemia for HDL-C [9]. Hypertension was diagnosed if blood pressure was over 130/85 mmHg, or already receiving antihypertensive treatment [9]. Hyperglycemia was described as previously diagnosed type 2 diabetes or fasting plasma glucose ≥ 5.56 mmol/L (100 mg/dL). Participants with 3 or more of 5 risk factors will be diagnosed with metabolic syndrome [9].

### Data analysis

Cox regression models were utilized to calculate the hazard ratio (HR) and 95% confidence interval (CI) for the correlation of MS and its components with HNC incidence risk. To compare the effect among MS components, we analyzed them as continuous variables to estimate HRs per standard deviation (SD) increase. In addition, we utilized restricted cubic splines for each MS component to explore their potential non-linear correlation with HNC risk. Further, to explore the combined effect between CRP and MS on the influence of HNC risk, we defined 4 risk levels according to MS or CRP levels with a cut-off point of 1.00 mg/dL [25]. And the HRs for HNC risk were calculated when comparing to the No MS plus CRP <1.00 mg/dL group.

The unadjusted model (model 1) was first conducted. Then, we adjusted age and gender in model 2. In model 3, ethnic, education, index of multiple deprivations, alcohol drinking status, smoking status, fruit and vegetable intake, physical activity, nonsteroidal anti-inflammatory drugs (NSAIDS) use, and CRP were further adjusted.

*P*<0.05 with two-tailed was considered as statistical significance. R software (version 3.6.1) was utilized for all statistical analysis.

## Results

### Baseline characteristics of participants

Among all participants, nearly one third of them were diagnosed as MS (*n* = 140346, 29.6%). As expected, the participants with MS have higher waist circumference, BMI, blood pressure, the plasma concentration of fasting glucose, TG, and CRP, but lower concentration of HDL-C. MS participants appeared to be older, to have a higher multiple deprivation index, and to have less physical activity. Table 1 showed the baseline characteristics.

**Table 1:** Baseline characteristics of participants according to MS in the UK biobank cohort

Metabolic syndrome (N)	No (***n*** = 334583)	Yes (***n*** = 140346)
**Average follow-up years, mean (SD)**	6.58 (1.23)	6.49 (1.30)
**Age at participation, mean (SD)**	55.5 (8.15)	58.3 (7.64)
**BMI, Mean (SD), kg/m**^**2**^	25.9 (3.86)	31.0 (4.90)
**Waist circumference, mean (SD), cm**	85.8 (11.3)	101 (12.1)
**HDL cholesterol, mean (SD), mmol/L**	1.55 (0.366)	1.22 (0.303)
**Triglycerides, mean (SD), mmol/L**	1.44 (0.777)	2.42 (1.19)
**Fasting glucose, mean (SD), mmol/L**	4.88 (0.671)	5.63 (1.87)
**SBP, mean (SD), mm Hg**	137 (19.7)	146 (17.9)
**DBP, mean (SD), mm Hg**	80.9 (10.6)	85.4 (10.3)
**Primary site of caner, N**		
Larynx	61	42
Tonsil	92	42
Oral cavity	181	111
Nasal cavity and sinuses	16	6
Oropharynx	14	7
Hypopharynx	20	4
Others	111	99
**Gender, N (%)**		
Female	188906 (56.5%)	66956 (47.7%)
Male	145677 (43.5%)	73390 (52.3%)
**Education level, N (%)**		
College or University degree	117845 (35.2%)	35265 (25.1%)
Other	162865 (48.7%)	70220 (50.0%)
unknown/missing	53873 (16.1%)	34861 (24.8%)
**Ethnicity, N (%)**		
White	315381 (94.3%)	131082 (93.4%)
Non-White	17645 (5.3%)	8492 (6.1%)
unknown/missing	1557 (0.5%)	772 (0.6%)
**Index of multiple deprivation quintile, N (%)**		
1th	61047 (18.2%)	20643 (14.7%)
2th	59990 (17.9%)	21823 (15.5%)
3th	58520 (17.5%)	23158 (16.5%)
4th	56301 (16.8%)	25571 (18.2%)
5th	52601 (15.7%)	29330 (20.9%)
Missing	46124 (13.8%)	19821 (14.1%)
**Smoking status, N (%)**		
Current	34587 (10.3%)	15915 (11.3%)
Previous	107678 (32.2%)	54704 (39.0%)
Never	190793 (57.0%)	68809 (49.0%)
Unknown/missing	1525 (0.5%)	918 (0.7%)
**Alcohol consumption, N (%)**		
Daily or almost daily	72332 (21.6%)	23976 (17.1%)
1–4 times a week	169384 (50.6%)	62723 (44.7%)
One to three times a month	35496 (10.6%)	17363 (12.4%)
Special occasions only or never	56642 (16.9%)	35901 (25.6%)
Unknown/missing	729 (0.2%)	383 (0.3%)
**Physical activity, N (%)**		
Low	44878 (13.4%)	26981 (19.2%)
Moderate	109466 (32.7%)	45595 (32.5%)
High	117170 (35.0%)	37074 (26.4%)
Unknown/missing	63069 (18.9%)	30696 (21.9%)
**Portions of fruit and vegetable intake, Mean (SD)**	4.66 (3.11)	4.52 (3.16)
**NSAIDS, N (%)**		
No	209520 (62.6%)	67714 (48.2%)
Yes	117629 (35.2%)	70322 (50.1%)
Missing	7434 (2.2%)	2310 (1.6%)
**CRP_C, N (%)**		
No	145619 (43.5%)	30015 (21.4%)
Yes	161311 (48.2%)	106964 (76.2%)
Missing	27653 (8.3%)	3367 (2.4%)

### Risk of HNC according to MS and its components

During an average follow-up of 6.5 years, we recorded 806 HNC cases. Overall, individuals with MS had no significant effect on risk of HNC compared to those without MS (HR, 1.05; 95%CI, 0.90–1.22). Five MS components (HR, 1.30; 95%CI, 0.92–1.84) led to a higher risk of HNC than 3 components (HR, 1.04; 95%CI, 0.88-1.24) and 4 components (HR, 1.00; 95%CI, 0.80–1.26) did, although no statistical differences were detected. Analysis of MS components reveals that individuals with dyslipidemia for TG (HR, 1.31; 95%CI, 1.31–1.51), hypertension (HR, 1.23; 95%CI, 1.02–1.48), and hyperglycemia (HR, 1.35; 95%CI, 1.14–1.61) had higher hazard for HNC (model 1). After being adjusted by age and gender (model 2), ethnic, education, index of multiple deprivations, alcohol drinking status, smoking status, fruit and vegetable intake, physical activity, NSAIDS use, and CRP (model 3), the association remained noticeably for hyperglycemia (HR, 1.22; 95%CI, 1.02–1.45). See details in Table 2.

**Table 2:** Risk of head and neck cancer according to MS and its components

	No of cases/person-years	Model 1		Model 2		Model 3	
		HR (95%CI)	***P***	HR (95%CI)	***P***	HR (95%CI)	***P***
**Presence of MS**							
No	495/ 2201184	Reference		Reference		Reference	
Yes	311/ 910374	**1.27[1.10, 1.47]**	**0.001**	**1.18[1.02, 1.36]**	**0.023**	1.05[0.90, 1.22]	0.560
**No. of MS components**							
0–2	483/2167132	Reference		Reference		Reference	
3	191/582520	**1.25[1.06, 1.48]**	**0.009**	1.14[0.96, 1.35]	0.126	1.04[0.88, 1.24]	0.645
4	95/283291	**1.26[1.01, 1.57]**	**0.043**	1.16[0.93, 1.45]	0.187	1.00[0.80, 1.26]	0.978
5	37/78614	**1.70[1.22, 2.38]**	**0.002**	**1.58[1.13, 2.21]**	**0.008**	1.30[0.92, 1.84]	0.132
**Center obesity**							
No	500/2065978	Reference		Reference		Reference	
Yes	304/1036668	1.10[0.95, 1.26]	0.211	**1.19[1.04, 1.38]**	**0.015**	1.04[0.90, 1.21]	0.592
**Dyslipidemia for TG**							
No	141/845849	Reference		Reference		Reference	
Yes	665/2262915	**1.31[1.13, 1.51]**	**<0.001**	1.06[0.91, 1.23]	0.461	0.95[0.81, 1.10]	0.472
**Dyslipidemia for HDL-C**							
No	301/1501095	Reference		Reference		Reference	
Yes	464/1438442	1.16[0.98, 1.39]	0.091	1.17[0.98, 1.39]	0.087	1.01[0.84, 1.22]	0.877
**Hypertention**							
No	528/2095502	Reference		Reference		Reference	
Yes	160/568287	**1.23[1.02, 1.48]**	**0.027**	1.02[0.84, 1.22]	0.874	1.00[0.82, 1.20]	0.973
**Hyperglycemia**							
No	529/2226708	Reference		Reference		Reference	
Yes	174/459089	**1.35[1.14, 1.61]**	**0.001**	**1.26[1.06, 1.5]**	**0.009**	**1.22[1.02, 1.45]**	**0.028**

Model 1: unadjusted

Model 2: age and gender-stratified model

Model 3: additionally adjusted for education, ethnic, index of multiple deprivation, alcohol consumption, smoking status, physical activity, fruit and vegetable intake, NASIDS use and CRP

### The non-linear associations for MS components and HNC risk

**Fig. 1:**
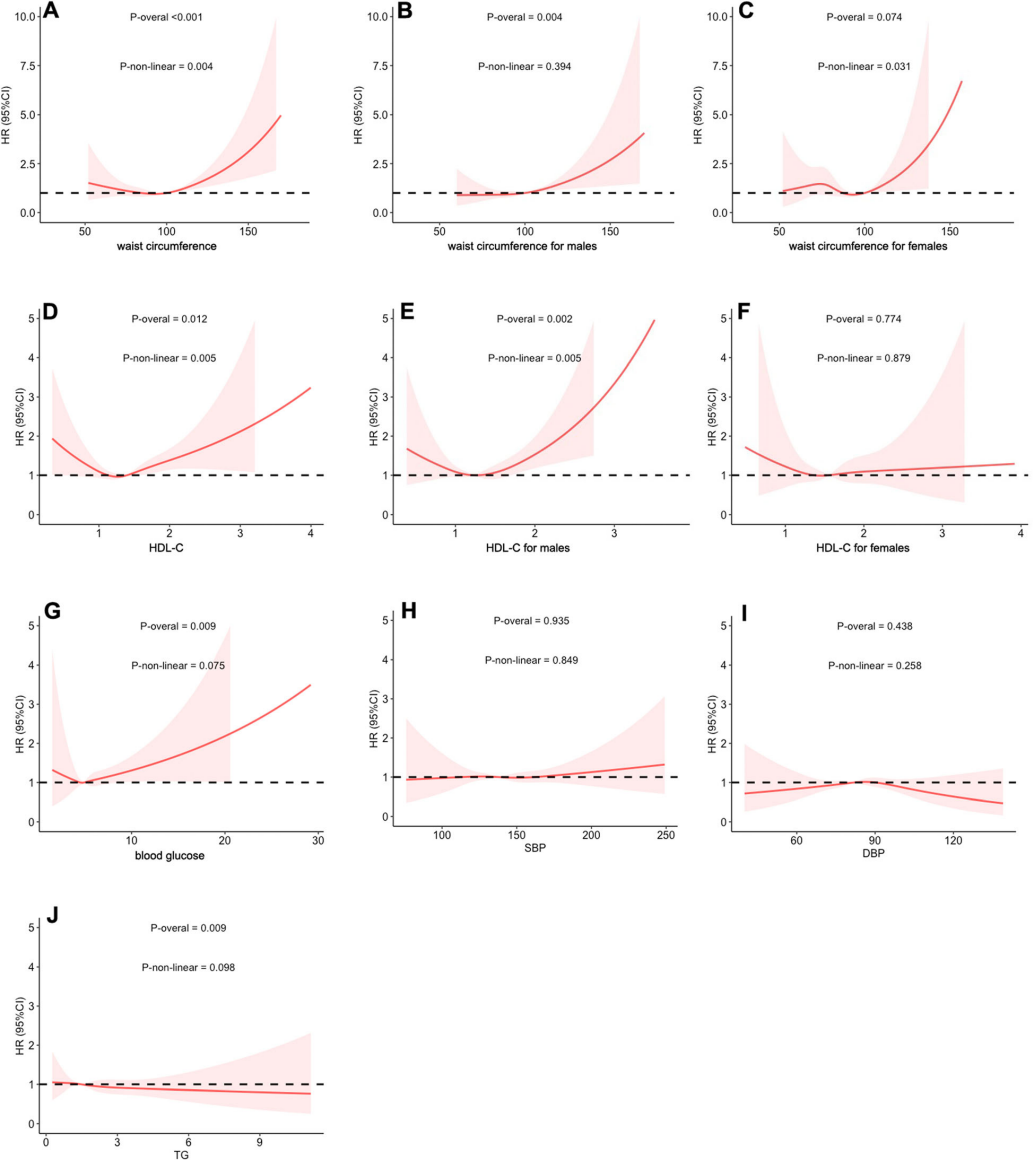
The non-linear effect between individual MS components and HNC risk. **A** Waist circumference, **B** waist circumference for males, **C** waist circumference for females, **D** HDL-C (high-density lipoprotein-cholesterol), **E** HDL-C for males, **F** HDL-C for females, **G** blood glucose, **H** SBP (systolic blood pressure), **I** DBP (diastolic blood pressure), **J** TG (triglyceride)

When assessing the non-linear effect between individual MS components and HNC risk, we observed a significant U-shaped association for waist circumference (Fig. 1A, p-non-linear=0.004) and HDL-C (Fig. 1D, p-non-linear=0.005) for the definition of abnormal waist circumference and HDL-C was sex-specific. Therefore, we analyzed their association for each gender, which showed that there was no non-linear relation between male waist circumference (Fig. 1B, p-non-linear=0.394), female HDL-C (Fig. 1F, p-non-linear=0.879), and HNC risk. However, a significant U-shaped association between female waist circumference (Fig. 1C, p-non-linear=0.031), male HDL-C (Fig. 1E, p-non-linear=0.005), and HNC risk was observed. Similarly, a U-shaped correlation for blood glucose (Fig. 1G, p-non-linear=0.075) and DBP (Fig. 1I, p-non-linear=0.258) was found but it was not significant. No relation was found for SBP (Fig. 1H, p-non-linear=0.849) and TG (Fig. 1J, p-non-linear=0.098).

### The linear associations for MS components related to HNC risk

We found that waist circumference (HR, 1.09; 95%CI, 1.01–1.18) was positively correlated with HNC risk in a fully adjusted COX model with MS components as a continuous linear term. However, HDL-C (HR, 1.07; 95%CI, 0.98–1.17) had no significant association with HNC risk. According to the definition criteria of MS and its components developed by the AHA/NHLBI, the critical values of abnormal waist circumference and HDL-C are sex-specific. Therefore, we further stratified them by gender and found that increased HNC risk was significantly related to male waist circumference (HR, 1.10; 95%CI, 1.02–1.20), but not related to female waist circumference (HR, 0.95; 95%CI, 0.82–1.10), male HDL-C (HR, 1.05; 95%CI, 0.97–1.15), and female HDL-C (HR, 1.08; 95%CI, 0.92–1.27).

Additionally, blood glucose (HR, 1.06; 95%CI, 1.01–1.12) was positively correlated with HNC risk in a fully adjusted model. However, no significant association was found for DBP (HR, 0.99, 95%CI, 0.93–1.07), SBP (HR, 1.03; 95%CI, 0.96–1.10), and TG (HR, 0.97; 95%CI, 0.91–1.04). See details in Table 3.

**Table 3:** Linear associations for MS components related to head and neck cancer risk

MS components	Model 1		Model 2		Model 3	
	HR (95% CI)	***P***	HR (95% CI)	***P***	HR (95% CI)	***P***
Waist circumference^a^	**1.43[1.34, 1.53]**	**<0.001**	**1.16[1.07, 1.25]**	**<0.001**	**1.09[1.01, 1.18]**	**0.032**
Waist circumference (male)	**1.19[1.10, 1.28]**	**<0.001**	**1.19[1.10, 1.28]**	**<0.001**	**1.10[1.02, 1.20]**	**0.019**
Waist circumference (female)^b^	0.98[0.85, 1.13]	0.788	0.98[0.85, 1.13]	0.796	0.95[0.82, 1.10]	0.490
HDL-C^a^	**0.78[0.72, 0.84]**	**<0.001**	1.02[0.93, 1.11]	0.723	1.07[0.98, 1.17]	0.098
HDL-C (male)^b^	1.01[0.93, 1.10]	0.801	1.01[0.93, 1.10]	0.783	1.05[0.97, 1.15]	0.243
HDL-C (female)^b^	1.02[0.87, 1.18]	0.830	1.02[0.88, 1.18]	0.821	1.08[0.92, 1.27]	0.329
Blood glucose^b^	**1.09[1.03, 1.15]**	**0.002**	**1.07[1.01, 1.13]**	**0.018**	**1.06[1.01, 1.12]**	**0.032**
Diastolic blood pressure^b^	**1.07[1.00, 1.05]**	**0.043**	0.99[0.92, 1.06]	0.716	0.99[0.93, 1.07]	0.848
Systolic blood pressure	**1.09[1.01, 1.17]**	**0.018**	1.03[0.96, 1.11]	0.415	1.03[0.96, 1.10]	0.451
Triglycerides (mmol/L)	**1.12[1.05, 1.20]**	**<0.001**	1.03[0.97, 1.11]	0.339	0.97[0.91, 1.04]	0.407

Model 1: unadjusted

Model 2: age and gender-stratified model

Model 3: additionally adjusted for education, ethnic, index of multiple deprivation, alcohol consumption, smoking status, physical activity, fruit and vegetable intake, NASIDS use and CRP

^a^MS component with sex-specific definition

^b^U-shaped association MS components related to head and neck cancer risk in non-linear spline models

### The further linear associations for MS components with U-shaped relation to HNC risk

Based on the U-shaped association results of non-linear spline models, we further divided the female waist circumference, male HDL-C, female HDL-C, blood glucose, and DBP into two sections with 93.16 cm, 1.26 mmol/L, 1.45 mmol/L, 4.70 mmol/L, and 83 mmHg, respectively, according to the lowest point of U-shaped curves to fit linear models. Too low waist circumference (<93.16 cm) showed no influence on female HNC risk (HR, 0.92; 95%CI, 0.78–1.08); but too high waist circumference (≥93.16 cm) was positively correlated with female HNC risk (HR, 1.47; 95%CI, 1.15–1.89). Interestingly, both too low (<1.26 mmol/L; HR, 0.88; 95%CI, 0.77–1.00) and too high HDL-C (≥1.26 mmol/L; HR, 1.19; 95%CI, 1.06–1.34) increased male HNC risk. There was no correlation between too low HDL-C (<1.45 mmol/L; HR, 0.91; 95%CI, 0.71–1.17) and too high HDL-C (≥1.45 mmol/L; HR, 1.10; 95%CI, 0.92–1.33) with female HNC risk. Too high blood glucose (≥4.70mmol/L) was positively correlated with HNC risk (HR, 1.10; 95%CI, 1.01–1.19); however, no relation was found for too low blood glucose (<4.70 mmol/L; HR, 0.96; 95%CI, 0.83–1.11). Both too low DBP (<83 mmHg; HR, 1.09; 95%CI, 0.98–1.21) and too high DBP (≥83 mmHg; HR, 0.99; 95%CI, 0.90–1.09) showed no significant influence on HNC risk. See details in Table 4.

**Table 4:** Linear associations for MS components with U-shaped relation to head and neck cancer risk

MS components	Model 1		Model 2		Model 3	
	HR (95% CI)	***P***	HR (95% CI)	***P***	HR (95% CI)	***P***
Waist circumference (female)						
<93.16 cm	0.94[0.80, 1.11]	0.469	0.94[0.80, 1.11]	0.471	0.92[0.78, 1.08]	0.311
≥93.16 cm	**1.42[1.11, 1.80]**	**0.005**	**1.41[1.11, 1.80]**	**0.005**	**1.47[1.15, 1.89]**	**0.003**
HDL-C (male)						
<1.26 mmol/L	**0.85[0.75, 0.97]**	**0.013**	**0.85[0.75, 0.97]**	**0.013**	**0.88[0.77, 1.00]**	**0.050**
≥1.26 mmol/L	**1.21[1.08, 1.36]**	**0.001**	**1.22[1.09, 1.36]**	**0.001**	**1.19[1.06, 1.34]**	**0.003**
HDL-C (female)						
<1.45 mmol/L	0.90[0.70, 1.14]	0.384	0.90[0.70, 1.14]	0.381	0.91[0.71, 1.17]	0.462
≥1.45 mmol/L	1.05[0.88, 1.27]	0.576	1.05[0.88, 1.27]	0.567	1.10[0.92, 1.33]	0.301
Blood glucose						
<4.70 mmol/L	0.93[0.80, 1.08]	0.330	0.97[0.84, 1.12]	0.674	0.96[0.83, 1.11]	0.577
≥4.70 mmol/L	**1.14[1.06, 1.22]**	**0.001**	**1.10[1.02, 1.19]**	**0.010**	**1.10[1.01, 1.19]**	**0.022**
Diastolic blood pressure						
<83 mmHg	**1.13[1.02, 1.25]**	**0.022**	1.07[0.96, 1.18]	0.221	1.09[0.98, 1.21]	0.121
≥83 mmHg	1.03[0.94, 1.14]	0.538	0.99[0.90, 1.10]	0.917	0.99[0.90, 1.09]	0.886

Model 1: unadjusted

Model 2: age and gender-stratified model

Model 3: additionally adjusted for education, ethnic, Index of multiple deprivation, alcohol consumption, smoking status, physical activity, fruit and vegetable intake, NASIDS use and CRP

### The relation between HNC risk and CRP and the combined effect of CRP and MS

We further explored the relation between HNC incidence risk and CRP, the combined effect of CRP and MS as well. Elevated CRP more than 1.00 mg/dL elevated the risk for HNC (HR, 1.21; 95%CI, 1.02–1.43) compared to it lower than 1.00 mg/dL (model 3). After evaluating the combined effect of CRP and MS, it was found that both no MS plus elevated CRP (HR, 1.22; 95%CI, 1.02–1.47) and MS plus elevated CRP (HR, 1.29; 95%CI, 1.05–1.58) participants had increased HNC risk compared to those without MS and CRP<1.00 mg/dL. But no joint effect between MS and CRP was detected (p-interaction=0.501). See details in Table 5.

**Table 5:** Risk of head and neck cancer according to CRP and the joint effect of MS and CRP

	No. of cases/person-years	Model 1		Model 2		Model 3	
		HR (95% CI)	***P***	HR (95% CI)	***P***	HR (95% CI)	***P***
CRP							
< 1.00 mg/dL	223/1155432	Reference		Reference		Reference	
≥ 1.00 mg/dL	531/1750092	**1.39[1.18, 1.62]**	**<0.001**	**1.44[1.23, 1.68]**	**<0.001**	**1.21[1.03, 1.42]**	**0.021**
Joint effect of MS and CRP	p-interaction=0.501						
No MS/CRP < 1.00 mg/dL	181/1027524	Reference		Reference		Reference	
No MS/CRP ≥ 1.00 mg/dL	327/1153876	**1.42[1.19, 1.72]**	**<0.001**	**1.45[1.21, 1.73]**	**<0.001**	**1.22[1.02, 1.47]**	**0.033**
MS/CRP < 1.00 mg/dL	42/127908	**1.50[1.07, 2.10]**	**0.018**	1.30[0.93, 1.82]	0.126	1.20[0.85, 1.68]	0.298
MS/CRP ≥ 1.00 mg/dL	204/596216	**1.58[1.29, 1.93]**	**<0.001**	**1.61[1.31, 1.96]**	**<0.001**	**1.29[1.05, 1.58]**	**0.017**

Model 1: unadjusted

Model 2: age and gender-stratified model

Model 3: additionally adjusted for education, ethnic, index of multiple deprivation, alcohol consumption, smoking status, physical activity, fruit and vegetable intake, and NASIDS use

## Discussion

This is a prospective cohort study involving 474,929 participants and 806 HNC cases. We observed that individuals with MS had no elevated incidence risk of HNC, and the risk did not elevate with the amount of MS components. Only hyperglycemia was independently correlated with HNC risk among all MS components. We also found that male waist circumference, female waist circumference (≥93.16 cm), male HDL-C (≥1.26 mmol/L), and blood glucose for each gender were positively correlated with HNC risk. CRP was positively correlated with an elevated incidence risk of HNC, and the risk was elevated in participants with MS, demonstrating MS, and CRP had joint effect on the risk of HNC. Our study comprehensively explored the correlation between MS and HNC risk in the general population and indicated an inflammatory mechanism for HNC development.

To date, there was only one study that exploring the effect of MS on the risk of HNC [26]. Stott-Miller et al. suggested a moderate inverse relation between MS and HNC. However, in our study, we observed no association between MS and HNC risk after adjusting several confounders. It is worth noting that Stott-Miller M’s study is a retrospective study, but our results are based on a prospective cohort study which has higher evidence level. Inadequate control of key confounding variables, including obesity, smoking time, and smoking intensity, in Stott-Miller M’s study may also lead to the observed reverse association.

Additionally, some researches have assessed the influence of MS on HNC component risk. Zucchetto et al. found no overall association between MS and nasopharyngeal carcinoma, but MS increased the risk of differentiated nasopharyngeal carcinoma [27]. However, a study conducted in South Korea by Sang-Yeon Kim claimed that MS was an independent risk factor for laryngeal cancer incidence [28]. Therefore, further analyses concerning the association between MS and HNC subgroups are meaningful in future studies.

In the present study, we observed that one MS component hyperglycemia was independently associated with increased HNC risk. Hyperglycemia is considered to be a critical factor in the pathophysiology of MS. Hyperglycemia, hyperinsulinemia, and insulin resistance are increasing proliferation, angiogenesis, and the destruction of DNA molecules by oxygen-active forms caused by excess glucose, cell migration, and apoptosis [29–31]. Previous studies suggested that MS elevated the incidence risk of cancer through the change of insulin receptors and activation of growth and transcription factors [32, 33]. Several studies have evaluated the influence of diabetes on HNC risk [26, 34–36]. Stott-Miller M [26] suggested that type II diabetes slightly decreased HNC risk. However, the other three studies revealed that diabetes increased HNC risk. Our study observed that blood glucose concentration was an independent risk factor for HNC development. Overweight/obesity is strongly correlated with glucose intolerance and type II diabetes [37, 38]; however, our study observed that central obesity was not an independent risk factor for HNC. Further analysis suggested that waist circumference was significantly correlated with HNC risk in a U-shaped manner. Moreover, the analysis stratified by gender was conducted in our study and revealed that the U-shaped association existed only between female waist circumference and HNC risk and the best cut-off point was 93.16 cm, which was firstly reported. Martina Recalde et al. concluded that non-linear BMI associations restricted to never smokers of head and neck, esophagus, stomach, trachea, bronchus, and lung cancers which strongly related to smoking [39]. Therefore, the U-shaped association only for female waist circumference very likely depends on the huge difference in smoking rates among males and females.

The relationship between HDL-C and cancer risk has not been fully understood yet. Most studies have showed that high level of HDL-C is protective against cancer [40]. However, we found that high HDL-C level increased the risk of HNC as well, which was also reported in prostate cancer [41, 42]. Just as some data are now challenging the long-hypothesized cardio-protective role of HDL, which show drugs that increase HDL do not decrease the risk of cardiovascular, which further question the health “benefits” of higher HDL [43, 44]. Therefore, it is meaningful to challenge the cancer-protective conception of high HDL-C. The mechanism by which high HDL-C increased cancer risk may be gene-associated. Two Mendelian randomization studies concluded that genetically raised HDL-C increased breast cancer risk [45]and HDL-C-raising variants in the target of cholesteryl ester transfer protein inhibitor gene were associated with increased breast cancer risk [46]. Yang et al. found that high HDL-C was positively associated with epidermal growth factor receptor (EGFR) mutation rate comparing with low HDL-C (59.0% vs. 35.6%) in lung cancer patients [47]. Simultaneously, the genomic instability or aneuploidy of EGFR gene has been identified as genetic alterations in each of the pathological stages of HNC [48], which may explain why too high HDL-C increase HNC risk. Further studies considering some additional confounders (such as oxidative stress or some unknown ones) and other biases are needed to clarify the intricate association between HDL-C and HNC risk. The concentration of triglycerides, diastolic, and systolic blood pressure had no independent influence on HNC risk.

Disorders of the inflammatory condition induced by MS may play an essential role the tumorigenesis. To date, no studies were available in exploring the influence of CRP on HNC risk. In the present study, our results indicated that CRP, a sensitive biomarker of inflammation in vivo, was independently correlated with an increased HNC incidence risk. Additionally, the HNC risk induced by MS plus CRP was further increased when compared with MS alone or elevated CRP alone. MS individuals had higher blood CRP, regardless of the diverse definition for MS and its components in different studies [49, 50]. This suggested that HNC development in MS individuals may attribute to the inflammatory system disruption. Therefore, it is critical to analyze the combined effects of MS and CRP during early intervention. Further researches are needed to verify these findings.

The primary benefit of this study is that the data are based on a prospective cohort study from the UK Biobank, with a verified follow-up time (average 6.5 years) and detailed measurement results. This allows potential confounding factors to adjust the correlation of interest simultaneously. In addition, we investigate the linear and non-linear relationship between all MS components and HNC risk, which has rarely been published in previous studies. Besides, we evaluated the interaction of MS and CRP with HNC, which may provide a pathological basis for MS tumorigenesis.

This study still has some limitations. First, as an observational study, although we have used complementary analytical methods to assess its epidemiological relationship steadily, we cannot assess the exact causal relationship between MS and HNC development. Second, because the MS component has only been measured once, it is impossible to assess these risk factors’ impact over time. Finally, due to the absence of histological information, it was limited to analyze the effects of MS on the HNC subtype.

## Conclusions

The intent of this study was to find the association between MS with HNC risk based on a prospective cohort study. Our study suggested no correlation between MS and HNC risk. However, waist circumference and blood glucose were the two predominant MS components that were independently associated with HNC risk. Different from the previous view that high HDL-C reduced the risk of some cancers, we concluded that high HDL-C increased the male HNC risk as well, suggesting the importance of controlling HDL-C in an appropriate range. There was no joint effect of MS and CRP in HNC tumorigenesis. This study may bring us new perception to study the pathological changes of HNC development.

## Data Availability

The data that support the findings of this study are available on request from the corresponding author. The data are not publicly available due to privacy or ethical restrictions.

## Abbreviations

HNC: Head and neck cancer
MS: Metabolic syndrome
HDL-C: High-density lipoprotein-cholesterol
CRP: C-reactive protein
DBP: Diastolic blood pressure
SBP: Systolic blood pressure
AHA/NHLBI: The American Heart Association/National Heart, Lung, and Blood Institute
BMI: Body mass index
TG: Triglycerides
HR: Hazard ratio
CI: Confidence interval
SD: Standard deviation
NSAIDS: Nonsteroidal anti-inflammatory drugs
EGFR: Epidermal growth factor receptor
